# On the WEDM of WNbMoTaZr*_x_* (*x* = 0.5, 1) Refractory High Entropy Alloys

**DOI:** 10.3390/e24121796

**Published:** 2022-12-08

**Authors:** Shunhua Chen, Kuang Xu, Weijie Chang, Yong Wang, Yucheng Wu

**Affiliations:** 1 School of Mechanical Engineering, Hefei University of Technology, Hefei 230009, China; 2 School of Materials Science and Engineering, Hefei University of Technology, Hefei 230009, China; 3 National-Local Joint Engineering Research Centre of Nonferrous Metals and Processing Technology, Hefei 230009, China

**Keywords:** WEDM, refractory high entropy alloy, cutting efficiency, surface roughness, statistical analysis, recast layer

## Abstract

As a potential candidate for the next generation of high-temperature alloys, refractory high entropy alloys (RHEAs) have excellent mechanical properties and thermal stability, especially for high-temperature applications, where the processing of RHEAs plays a critical role in engineering applications. In this work, the wire electrical discharge machining (WEDM) performance of WNbMoTaZr*_x_* (*x* = 0.5, 1) RHEAs was investigated, as compared with tungsten, cemented carbide and industrial pure Zr. The cutting efficiency (CE) of the five materials was significantly dependent on the melting points, while the surface roughness (Ra) was not. For the RHEAs, the CE was significantly affected by the pulse-on time (ON), pulse-off time (OFF) and peak current (IP), while the surface roughness was mainly dependent on the ON and IP. The statistical analyses have shown that the CE data of RHEAs have relatively-smaller Weibull moduli than those for the Ra data, which suggests that the CE of RHEAs can be tuned by optimizing the processing parameters. However, it is challenging to tune the surface roughness of RHEAs by tailoring the processing parameters. Differing from the comparative materials, the WEDMed surfaces of the RHEAs showed dense spherical re-solidified particles at upper recast layers, resulting in larger Ra values. The proportion of the upper recast layers can be estimated by the specific discharge energy (SDE). Following the WEDM, the RHEAs maintained the main BCC1 phase, enriched with the W and Ta elements, while the second BCC2 phase in the Zr1.0 RHEA disappeared. Strategies for achieving a better WEDMed surface quality of RHEAs were also proposed and discussed.

## 1. Introduction

As proposed in 2004, high entropy alloys (HEAs) have attracted great research attention due to their excellent mechanical properties, corrosion and radiation resistance, and thermal stability [1,2,3,4]. Differing from conventional alloys with principal elements, HEAs are also known as multi-principal alloys, which consists of at least five principal elements in equal or near-equal atomic ratios [5,6]. Inspired by the necessities for high-temperature applications, refractory high entropy alloys (RHEAs) have subsequently been developed in 2010, containing at least four of the nine refractory elements (Ti, V, Cr, Zr, Nb, Mo, Hf, Ta, W) [7,8,9]. With outstanding mechanical properties at high temperature, RHEAs are considered as desirable candidate materials to substitute Ni-based superalloys in the future, for high-temperature applications [10,11,12]. With the application potential for aerospace, mining machinery, and nuclear fusion reactors [13,14,15], the phase formation, microstructure, and mechanical properties of RHEAs, such as strength, plastic deformation mechanisms, and fracture behavior, have been investigated extensively [16]. However, RHEAs are classified as difficult-to-cut materials, due to their high strength and hardness which are superior to conventional alloys. The processing of RHEAs plays a key role in their practical applications in industry. Some studies about the cutting of the W alloys have shown that the cutting tools showed severe wear, due to the high hardness [17]. Moreover, the brittle removal and the grain collapse are easy to occur during the cutting process, resulting in a higher surface roughness and poor surface integrity. The cutting of Ti alloys, which are widely applied in aerospace industries, is also very challenging. The high strength and low thermal of the Ti alloys often cause a high cutting temperature, which significantly influences the machined surface quality and tools lifetime [18]. Studies on the conventional machining of HEAs have shown that although a good surface quality can be achieved, the lifetime of cutting tools was significantly reduced due to numerous cutting heat and severe wear [19,20,21]. While for the RHEAs with a higher strength and hardness, how to process RHEAs is more challenging but important for their engineering applications.

As a well-known non-traditional machining technology with non-contact force [22,23], wire electrical discharge machining (WEDM) has been widely employed to process difficult-to-cut materials, including bulk metallic glasses (BMGs), ceramics, and carbides [24,25,26,27,28]. For example, Chang et al. [28] studied the effects of WEDM processing parameters on the surface roughness and cutting efficiency of BMGs, where a good surface quality was achieved at a higher cutting efficiency, based on the optimization of the processing parameters. The mechanisms for the change of the parent elements during the sparking were discussed, and the findings have validated the feasibility of processing the BMGs using the WEDM technique. Ming et al. [29] investigated the effects of the processing parameters and material composition on the WEDM performance of the BN-AlN-TiB_2_ composite ceramics, and a finite element model was established, to predict the WEDM performance. The results have shown that such a model has a higher accuracy in the prediction of the kerf width, and the WEDM performance was mainly affected by the discharge energy. Shayan et al. [25] reported the effects of the processing parameters of the dry WEDM on the cutting velocity, surface roughness, and oversize of the cemented tungsten carbide (WC-Co). A back-propagation neural network (BPNN) model was proposed to predict and optimize the machining performance at different processing parameters. For RHEAs with refractory elements, recent studies have shown that the surface quality can be improved by changing the machining modes and selecting the appropriate wire electrode [30,31]. However, the WEDM performance of RHEAs under varying electrical parameters has not been fully explored. The relationship between the WEDMed surface morphology and the processing parameters also needs to be further revealed.

In this work, the effects of the electrical parameters, including the pulse-on time (ON), pulse-off time (OFF), peak current (IP), servo voltage (SV), pulse type (GP), and barrel running frequency (WS), on the WEDM performance of WNbMoTaZr*_x_* (*x* = 0.5, 1) RHEAs were investigated, and compared with three conventional materials (tungsten, cemented carbide, and industrial pure zirconium). Strategies to achieve a better WEDM performance of RHEAs were also proposed and discussed.

## 2. Materials and Methods

### 2.1. Workpiece Materials

WNbMoTaZr*_x_* (*x* = 0.5 and 1, denoted as Zr0.5 and Zr1.0, respectively) RHEAs were prepared by vacuum arc melting under a Ti-gettered high-purity argon atmosphere, using raw metals with a purity higher than 99.95% [32]. Each alloy ingot was re-melted 12 times, to ensure the homogeneity. For comparison, the WEDM performance of industrial pure zirconium (denoted as Zr702), tungsten, and cemented carbide (YG8) was also examined. The composition of the five materials is shown in Table 1. Since the thermodynamic properties of workpiece materials have a significant impact on the WEDM performance, the thermodynamic parameters of the five workpiece materials including the melting point and thermal conductivity are listed in Table 2. The thermal conductivity of the Zr0.5 and Zr1.0 RHEAs was obtained using the NETZSCH LFA457 MicroFlash, and the melting points were theoretical values from Ref. [32].

### 2.2. WEDM Processing

**Figure 1:**
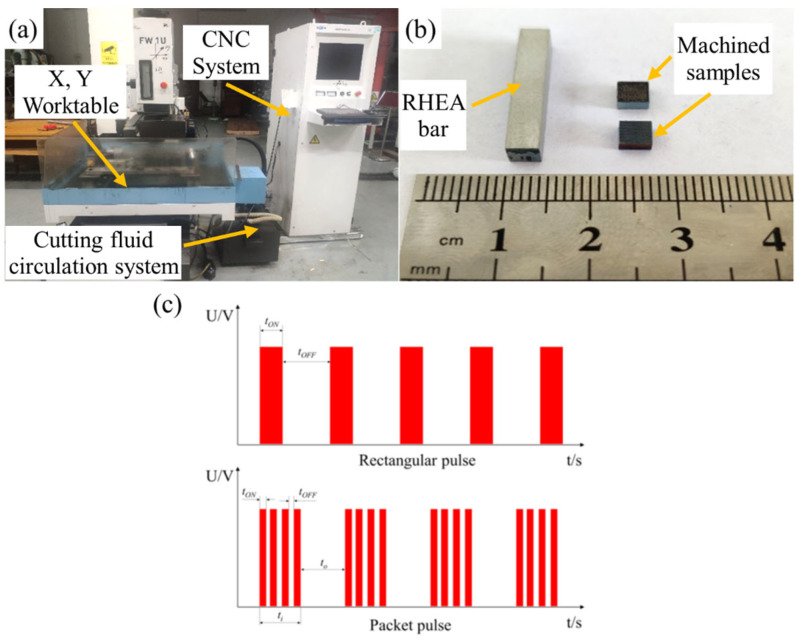
(**a**) WEDM machine tool; (**b**) RHEA bar before the WEDM examinations and the machined samples; (**c**) Pulse waveform diagram of the machine tool.

The WEDM experiments were conducted on an AgeCharmilles FW1U WEDM machine tool, as shown in Figure 1a, where the molybdenum wire electrode with a diameter of 0.18 mm and water-based cutting fluids were used. Bars with a cross-section dimension of 4 mm × 4 mm were cut from the ingot for the subsequent WEDM performance examinations. The bars were then cut along the cross-section to the shape of 4 mm × 4 mm × 2 mm, as shown in Figure 1b. The cutting efficiency (CE) is expressed by the following equation:

(1) CE=S/t

where *S* (mm^2^) is the cross-section area and *t* (min) is the processing time. In order to compare the WEDM performance of RHEAs with conventional materials, orthogonal experiments with six parameters and three levels were designed. The detailed processing parameters, including the pulse-on time (ON), pulse-off time (OFF), peak current (IP), servo voltage (SV), pulse type (GP), and barrel running frequency (WS), are given in Table 3, where the GP code 00 represents the rectangular pulse, and the GP codes 01 and 02 represent two different packet pulses, as shown in Figure 1c. To further examine the effects of the processing parameters (ON, OFF, IP, and SV) on the WEDM performance of RHEAs, the single-factor WEDM experiments were designed subsequently, based on the orthogonal testing results. After WEDM, the surface roughness (Ra) was measured using a roughness tester, where six data were collected and the average value was used for the subsequent analyses. The phase structure of the WEDMed surfaces was characterized by a PANalytical X-Pert PRO MPD X-ray diffractometer. The WEDMed surface morphology was inspected on a Gemini 500 scanning electron microscope (SEM) equipped with an energy dispersive spectroscope (EDS).

## 3. Results and Discussion

### 3.1. WEDM Results, Based on the Orthogonal Experiments

#### 3.1.1. Cutting Efficiency (CE)

**Figure 2:**
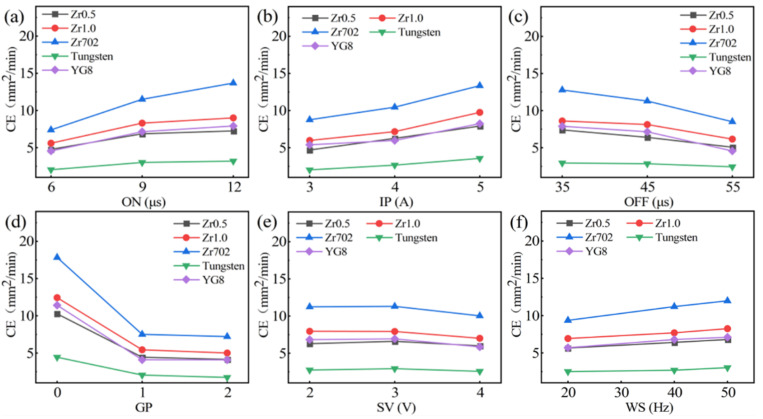
The variation of the cutting efficiency (CE) on the change of the processing parameters during the WEDM of the five workpiece materials (SN ratio): (**a**) ON; (**b**) IP; (**c**) OFF; (**d**) GP; (**e**) SV; and (**f**) WS.

The WEDM cutting efficiency of the five workpiece materials are shown in Table 4. The CE of the workpiece materials varied significantly among the different groups, and the highest CE was obtained in group 8 specimens for the five workpiece materials. Based on the CE results, the change of the CE on the variation of the processing parameters was further analyzed. As shown in Figure 2, the CE of the five workpiece materials increased with the increase of the IP and ON. This is due to the fact that the increase of the IP and ON can enhance the discharge energy of a single pulse, which benefits the melting and removal of the workpiece materials, resulting in the improvement of the CE. On the contrary, with the increase of the OFF (Figure 2c), the decrease of the CE can be attributed to the decreased discharge energy per unit time. As shown in Figure 2d, when the GP transformed from the rectangular pulse (00) to packet pulses I (01) and II (02), the CE decreased significantly but with similar values for the two packet pulses (01 and 02). This resulted from the differences between the waveforms. As shown in Figure 1c, the packet pulses were derived from the rectangular pulse, and there is a longer pulse-off time (*t*_0_) between each set of pulses. The discharge energy of a single pulse is the main parameter determining the size of the discharge craters, which affects the material removal rate (*MRR*) [27,36]. The discharge energy of a single pulse can be determined by the discharge voltage, current, and the pulse-on time, as described by the equation [28]:

(2) $$W_{o}=\int_{0}^{t_{i}}u\left(t\right)i\left(t\right)dt$$

where *W*_o_ is the discharge energy of a single pulse, *u(t)* is the instantaneous discharge voltage, *i(t)* is the instantaneous discharge current, and *t*_i_ is the continuous discharge time. It can be seen that the discharge energy *W*_o_ increased with the increase of *u(t)*, *i(t)*, and *t*_i_. With the change of the GP from a rectangular pulse (00) to packet pulses (01 and 02), the discharge energy of a single pulse keeps unchanged, however, the number of discharge pulses per unit time was reduced, due to the existence of a long pulse interval *t*_0_ between each set of pulses, leading to the decrease of the discharge energy and the resultant CE. While for the other two processing parameters (SV and WS), no significant change in the variation of the CE was observed.

The analysis of variance (ANOVA) for the CE of the five workpiece materials are listed in Appendix A Table A1. The contributions of the processing parameters on the CE of five materials are highly in line with the results in Figure 2. The GP had the most significant contribution for all materials, where the value reached 64.69%, 63.61%, 60.93%, 64.11%, and 65.22% for Zr0.5, Zr1.0, Zr702, tungsten, and YG8, respectively. Such contribution agrees well with the significant decrease of the CE in Figure 2d. Except the GP, ON, and IP were the two main processing parameters which contributed to the CE of the WEDM process, followed by the OFF, for most of the workpiece materials.

#### 3.1.2. Surface Roughness (Ra)

**Figure 3:**
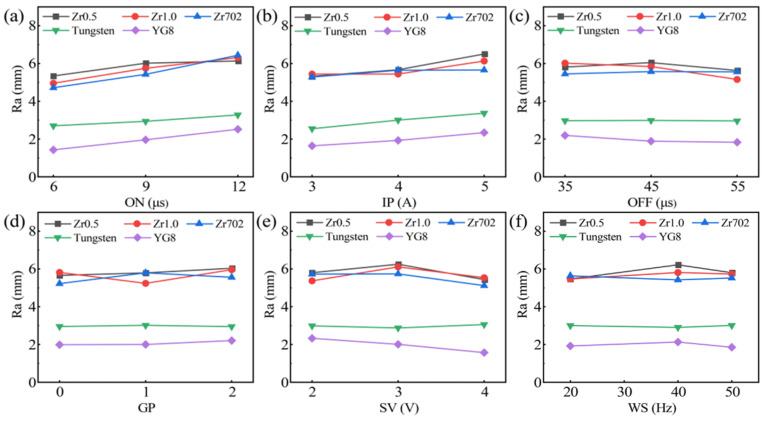
The variations of the surface roughness (Ra) on the change of the processing parameters during the WEDM of the five workpiece materials (SN ratio): (**a**) ON; (**b**) IP; (**c**) OFF; (**d**) GP; (**e**) SV; and (**f**) WS.

The surface roughness data (Ra) of the WEDMed surfaces are shown in Table 5, and the variations on the change of the processing parameters are shown in Figure 3. As compared with the CE data, the surface roughness (Ra) data have smaller orders of variations. Nevertheless, the slightly increasing trends of the Ra data with the increase of the ON and IP have also been observed, while the change of other parameters has no significant effect on the variation of the surface roughness. According to the ANOVA of the five workpiece materials, as shown in Appendix A Table A2, the contributions of the processing parameters on the surface roughness of the five materials are different. The ON and IP are the two main contributors for the Zr0.5, Zr1.0, and tungsten, while the ON and SV play a more significant role for Zr702 and YG8. Differing from the contributions for the CE (Table A1), the contributions of the processing parameters for the surface roughness of RHEAs were more evenly distributed. For example, for the Zr1.0 RHEA, five parameters (OFF, GP, SV, IP and ON) have contributions ranging from 9.71% to 36.63%. This indicates the relatively-smaller variations of the surface roughness on the change of the processing parameters, similar to the findings, as shown in Figure 3. The detailed variations of the CE and Ra data will be discussed later in a subsequent section.

**Figure 4:**
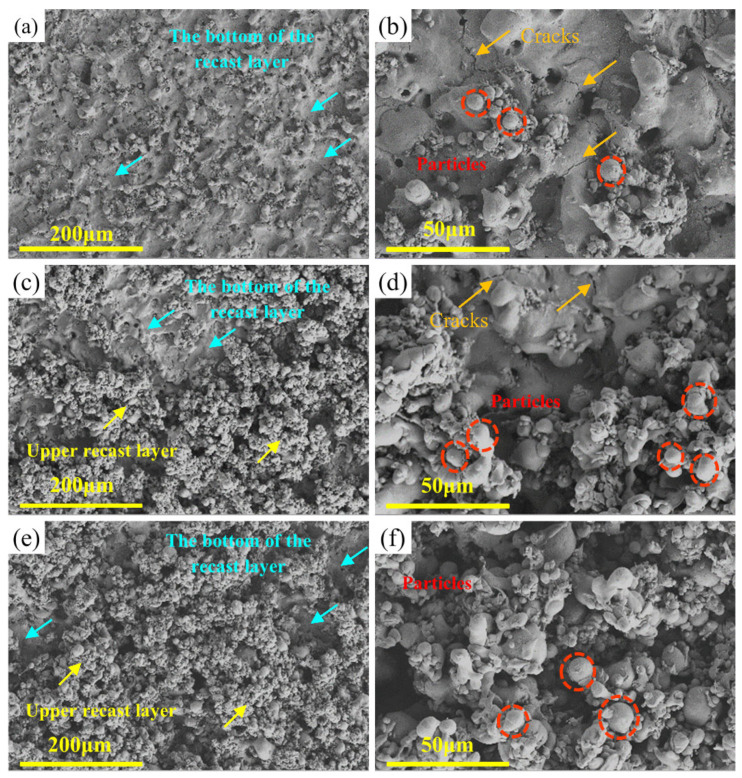
SEM images showing the WEDMed surface morphologies of the Zr1.0 RHEA under different conditions: (**a**,**b**) group 9; (**c**,**d**) group 13; and (**e**,**f**) group 8, where the upper recast layer consisting of particles, the bottom of the recast layer, and the cracks, are indicated.

In order to better characterize the variation of the surface roughness, the WEDMed surface morphology of the specimens of the typical Zr1.0 RHEAs in groups 9, 13, and 8, with average surface roughness (Ra) values of 4.18 μm, 6.14 μm, and 7.27 μm, respectively, was given in Figure 4. After WEDM, the significant solidification of the melted alloys was observed, where a large number of re-solidified particles were found, especially for the specimens in group 13 and group 8. These dense re-solidified particles formed the upper recast layer, as indicated in Figure 4c,e, which was related to the higher surface roughness values. The machined surface of the group 9 specimen was mainly composed of the bottom of the recast layer, which had the lowest surface roughness (Figure 4a). The increased region of the upper recast layer, composed of the re-solidified particles from group 13 to group 8 specimens resulted in the increased surface roughness. By examining the formation of the recast layer with the re-solidified particles at higher magnifications (Figure 4b,d,f), the particles tend to form clusters, and the upper layer of the recast layer consists mainly of clusters of re-solidified particles. The formation of such clusters and re-solidified particles results from the sparking process for the melting and the removal of the melted workpiece material. The melting and removal of the workpiece materials were related to the corresponding consumed discharge energy during the WEDM. The concept of the specific discharge energy (SDE) was proposed to estimate the machining characteristics [37], which was defined as the real consumed discharge energy, to remove a unit volume of materials. The SDE can be expressed as:

(3) $$SDE=\frac{T_{\mathrm{on}}\times IP\times SV}{\left(T_{\mathrm{on}}+T_{\mathrm{off}}\right)\times MRR}$$

where *T*_on_ is the pulse-on time, *T*_off_ is the pulse-off time, and *MRR* is the material removal rate. The output energy of the WEDM tool was not entirely employed for the removal of the materials, and the partial energy was consumed by the light, noise, dielectric, and electrode wire [38]. Thus, the actual discharge energy consumed in the removal of the materials can be expressed as:

(4) $$SDE^{\prime }=\frac{T_{\mathrm{on}}\times IP\times SV}{\left(T_{\mathrm{on}}+T_{\mathrm{off}}\right)\times MRR}f_{c}$$

where *f*_c_ is the energy distribution ratio of the workpiece materials. Previous studies showed that *f*_c_ was the function of the current, the pulse-on time, and the material properties [39,40]. In this work, the cutting efficiency (CE) was used to estimate the processing performance. The relationship between *MRR* and the CE can be given as:

(5) $$MRR=CE\times W_{k}$$

where *W*_k_ is the kerf width. With an electrode wire diameter of 0.18 mm, the kerf width of each specimen was also approximately estimated as 0.18 mm, and the simplified SDE was expressed as:

(6) $$SDE_{s}=\frac{T_{\mathrm{on}}\times IP\times SV}{0.18\left(T_{\mathrm{on}}+T_{\mathrm{off}}\right)CE}f_{c}$$

Based on the smaller parameter ranges in the orthogonal experiments and the simplification of the calculation process, the *f*_c_ was approximately regarded as a constant value. The consumed energy of the workpiece materials was therefore calculated as 3.27 *f*_c_, 1.27 *f*_c_, and 0.97 *f*_c_ for the specimens in groups 9, 13, and 8, respectively. A higher calculated value indicates that the melted workpiece materials have a higher discharge energy, resulting in larger explosive forces and a lower viscosity. Therefore, the melted droplets can be removed more easily, and further reduce the upper recast layer, composed of re-solidified particles, resulting in a lower surface roughness. Moreover, a few cracks on the bottom of the recast layer were observed, as shown in Figure 4b,d. This may result from the rapid cooling of the machined surface or high residual stress.

### 3.2. Comparison of the WEDM Performance

#### 3.2.1. Cutting Efficiency (CE)

**Figure 5:**
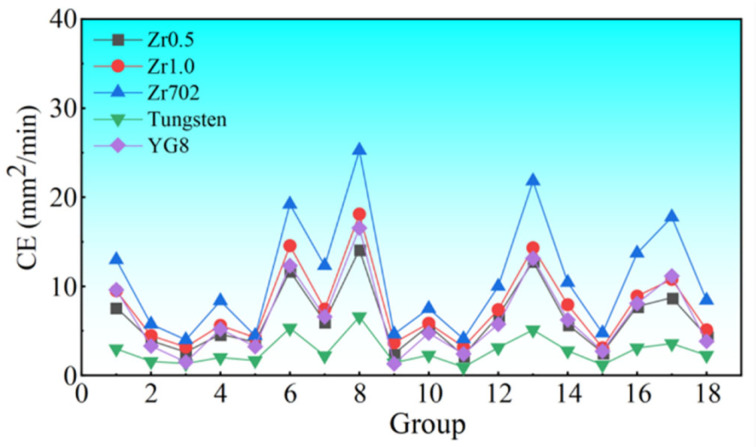
Comparison of the CE under different processing parameters.

The CE of the five workpiece materials was compared, as shown in Figure 5. Although the five workpiece materials have similar variation trends on the change of groups, the CE of each material exhibits obvious differences. Among the five materials, Zr702 exhibited the highest CE, followed by Zr0.5, Zr1.0 RHEA, and YG8, and tungsten had the smallest CE. The variations of the CE were then characterized using a statistical analysis. Overall, a higher CE was observed in the workpiece materials with a lower melting point, where the tungsten with a melting point of 3695 K has the lowest average CE of 2.74 mm^2^/min, and the Zr702 with a melting point of 2128 K, had the highest CE of 10.86 mm^2^/min. While the other three workpiece materials, with melting points ranging from 2952 K to 3196 K, have an average CE ranging from 6.29 mm^2^/min to 7.63 mm^2^/min. Thus, the difference of the CE among the different workpiece materials was mainly attributed to the differences in the melting point. During the WEDM, the workpiece material was removed by the melting and throw-out of melted workpiece material. When the same amounts of materials are removed, more energy is required, to melt and throw the workpiece material with a higher melting point. Therefore, high melting point materials generally have a lower CE.

**Figure 6:**
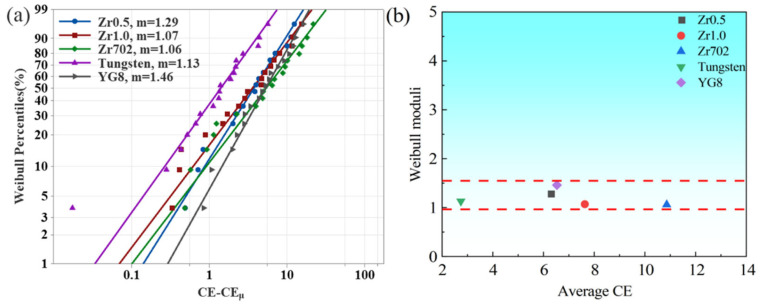
(**a**) The fitting results of the three-parameter Weibull distribution of the CE, and (**b**) the relationship between the Weibull moduli and the average CE of the five workpiece materials.

The variation of the CE was then fitted via the three-parameter Weibull distribution, as shown in Figure 6. The cumulative probability of the CE, based on the three-parameter Weibull distribution can be calculated by the equation:

(7) $$P_{e}=1-\exp\left[-{\left(\frac{CE-CE_{\mu }}{CE_{0}}\right)}^{m}\right]$$

where *P*_e_ is the probability of the corresponding value, *CE*_μ_ is the cut-off value and *CE*_0_ is the scale parameter. *m* is the shape parameter, i.e., the Weibull modulus, which can be expressed by the linearizing Equation (7) to:

(8) $$\ln\left[\ln\left(\frac{1}{1-P_{e}}\right)\right]=m\ln\left(CE-CE_{\mu }\right)-m\ln\left(CE_{0}\right)$$

As shown in Figure 6, it can be seen that all the five materials have small Weibull moduli from 1.06–1.29. For three parameter Weibull distribution, a larger Weibull modulus represents a smaller variation of the data, i.e., the better uniformity of the data distribution. Here, the smaller Weibull moduli of the CE suggest large variations among the different groups, agreeing well with the results, as shown in Figure 5. As shown in Figure 6b, although the average CE of five materials had a larger difference, all the Weibull moduli have relatively smaller values, around 1, indicating the larger variation of the CE for the five materials. Thus, with large variations on the change of the different processing parameters, the CE can then be tuned, based on the optimization of the processing parameters.

#### 3.2.2. Surface Roughness (Ra)

**Figure 7:**
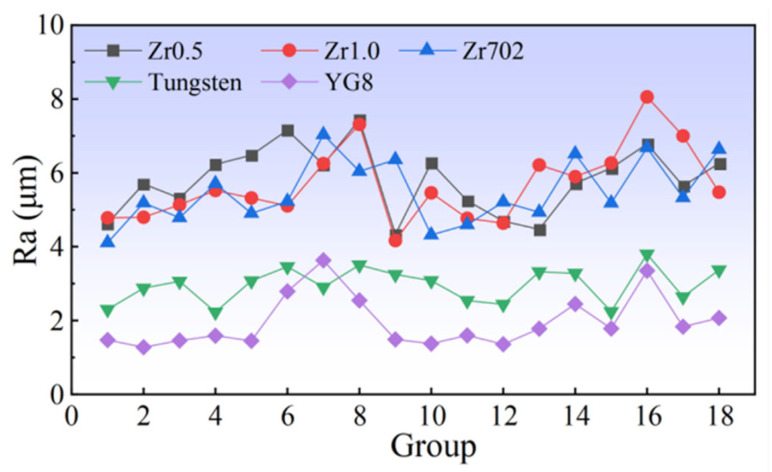
Comparison of the surface roughness (Ra) under different processing parameters.

**Figure 8:**
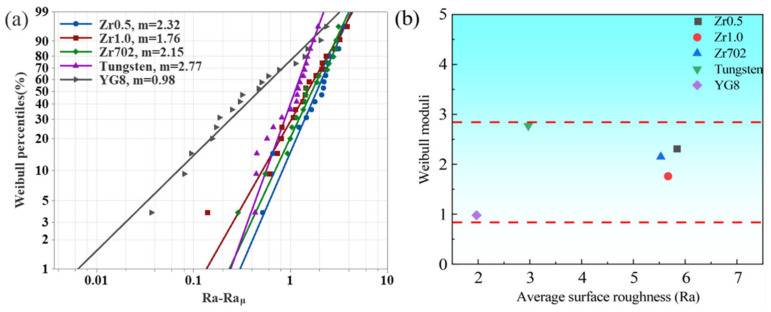
(**a**) The fitting result of the three-parameter Weibull distribution of the CE, and (**b**) the relationship between the Weibull moduli and the average CE of the five workpiece materials.

The comparison of the surface roughness is shown in Figure 7. Differing from the results of the CE, the variation trend of surface roughness for the five workpiece materials has a significant difference. The surface roughness values of Zr0.5, Zr1.0, and Zr702, are higher than that of the pure tungsten and YG8. The Zr702 has the lowest average surface roughness (Ra) of 1.97 μm, and the tungsten has an average Ra value of 2.97 μm. While the other three workpiece materials have a similar average Ra value ranging from 5.53 μm to 5.83 μm. Although the CE was dependent on the melting point of the workpiece materials, the melting point had a limited effect on the surface roughness of the five materials. The surface roughness data were also analyzed using the three-parameter Weibull distribution given by Equations (7) and (8). According to Figure 8a, the Weibull moduli for the surface roughness data of Zr0.5, Zr1.0, Zr702, and tungsten, are larger than that of the CE, which implies that the surface roughness data of these materials have the higher uniformity than the CE. As shown in Figure 8b, the Zr1.0, Zr0.5, and Zr702 have a similar average surface roughness and similar Weibull moduli around 2. While the tungsten has the largest Weibull modulus, indicating a higher uniformity of the data. Differing from the CE results with relatively larger variations, the surface roughness of RHEAs exhibit ahigher uniformity but relatively larger values. Thus, during the WEDM, it is challenging to tune the surface roughness of the RHEAs by changing the processing parameters, where more attention may be paid to the melting and removal mechanisms of the RHEAs during sparking.

**Figure 9:**
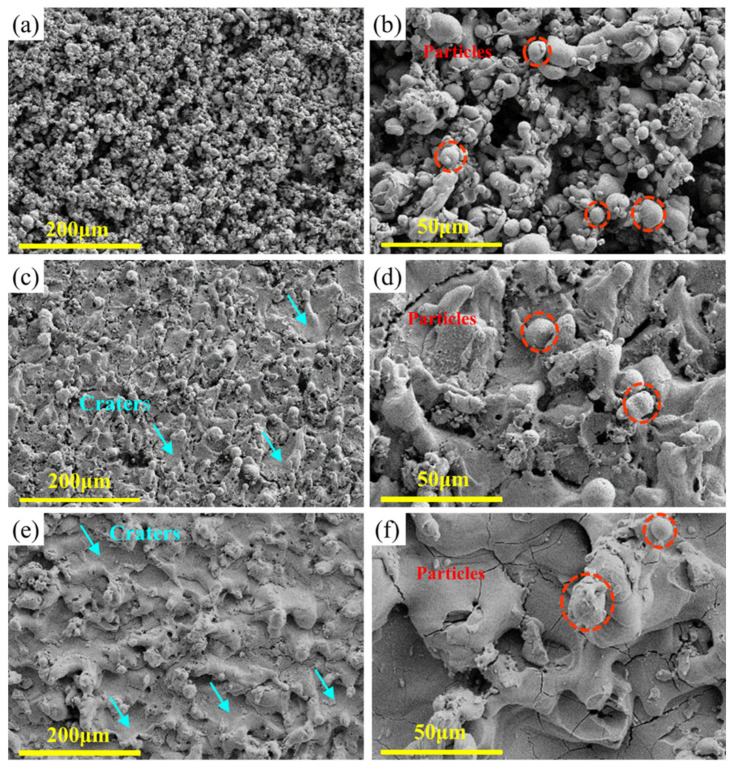
SEM images showing the WEDMed surface morphologies of the Zr0.5 RHEA (**a**,**b**), tungsten (**c**,**d**), and Zr702 (**e**,**f**) in group 8, where the spherical particles at the upper recast layer and the craters at the bottom of the recast layer are indicated.

The surface morphology of the typical Zr0.5 RHEA, tungsten, and Zr702 specimens, with Ra of 7.50 μm, 6.30 μm, and 3.59 μm, respectively, in group 8 is shown in Figure 9. Similar to the surface morphology of the Zr1.0 RHEA (Figure 4e,f), the Zr0.5 RHEA had a dense distribution of clusters and particles (Figure 9a,b), which covered the WEDMed surface, resulting in a higher surface roughness. For tungsten (Figure 9c,d), although the spherical melted particles were also observed, the number of particles was much smaller than that for the RHEAs, and the clusters of particles were not observed. The bottom of the recast layer can be clearly observed, where many cracks, similar to the Zr1.0 RHEA (Figure 4d), were found, resulting in a lower surface roughness. This indicates that the amount of melted tungsten thrown out during sparking was larger than that of the Zr0.5 RHEA. According to Equation (6), the calculated values of the Zr0.5 RHEA and tungsten are 1.24*f*_c_ and 2.67*f*_c_, respectively. Despite the higher melting point, the energy actually used to process a unit quantity of W was much higher than that of the Zr0.5 RHEA, resulting in larger explosive forces and a low viscosity for the melted liquids. Most of the melted liquids were therefore removed during sparking. As shown in Figure 9d,e, the WEDMed surface of Zr702 had much larger sizes of particles, craters, and ridges. Due to the lowest melting point, the Zr702 workpiece material was easier to be melted. The larger sizes of craters and ridges were also more likely to form, resulting in the high surface roughness data.

### 3.3. Microstructure and the Element Variation of the WEDMed Surfaces of RHEAs

**Figure 10:**
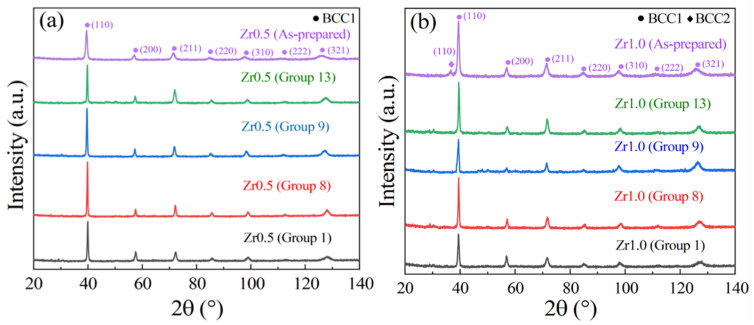
XRD patterns of the as-prepared RHEAs and the WEDMed surfaces of the Zr0.5 (**a**) and Zr1.0 (**b**) RHEAs, respectively.

During WEDM, a large amount of heat generated, which may result in the phase transformation on the WEDMed surfaces. For example, Rahman et al. [41] investigated the WEDM performance of the Ti-6Al-4V ELI alloy, and found that the phase transformed from α to α′ and rutile-TiO_2_, in the recast layer. Huang et al. [42] studied the effect of the WEDM on the surface properties of P/M high-speed steel, and found the martensitic transformation on the surface recast layer, due to rapid quenching cycles. The microstructure and element variation of the WEDMed surfaces of RHEAs are critical indicators of the WEDMed surface quality, which has a significant effect on the WEDM performance. Therefore, in this work, the phase structure of the recast layers of RHEAs was also examined using an X-Ray diffraction (XRD) analysis. The XRD patterns of the typical Zr0.5 and Zr1.0 RHEA specimens in groups 1, 8, 9 and 13 are shown Figure 10. For the Zr0.5 RHEA with a mainly BCC1 phase (Figure 10a), the WEDMed surfaces mainly consist of a similar phase structure, i.e., the BCC1 phase for all of the conditions. While for the Zr1.0 RHEA with two BCC phases in the as-prepared state, the WEDMed surface exhibited the main BCC1 phase, and the second BCC2 phase was eliminated during sparking (Figure 10b). Additionally, although a small number of low-intensity peaks corresponding to unknown phases appeared, their contents were almost negligible, based on the intensity of their diffraction peaks. As demonstrated in Ref. [32], the BCC2 phase is enriched with Zr and Nb elements with a relatively lower melting point, while the BCC1 phase is enriched with the W and Ta elements with a higher melting point. Differing from the significant carbonization effect of the BMGs during the WEDM [28], the BCC1 phase with high melting point elements (W and Ta) was not affected by sparking. While the BCC2 phase with Zr and Nb in the Zr1.0 RHEA disappeared.

The variations of the parent elements (W, Nb, Mo, Ta, and Zr) on the WEDMed surfaces of the Zr0.5 and Zr1.0 RHEAs were inspected using point EDS analysis. For two typical groups of specimens, after the WEDM, asignificant decrease of the Zr content was observed for both the Zr0.5 and Zr1.0 RHEAs, and the Zr1.0 RHEA with a higher Zr content had an increased loss of the parent Zr element. The XRD results have suggested that the content of the BCC2 phase was reduced or even removed during the WEDM process. Moreover, previous findings have shown that the BCC2 phase was enriched with the Zr and Nb elements for the as-prepared Zr1.0 RHEA [32]. This suggests that the disappearance of the BCC2 phase is closely related to the reduction of the Zr content. These findings provide explanations for the disappearance of the BCC2 phase, based on the XRD results (Figure 10b). Moreover, the present findings have shown that the original microstructure of the RHEAs had a significant effect on the WEDM process, where more attention should be paid to the underlying WEDM mechanisms, in the future.

**Figure 11:**
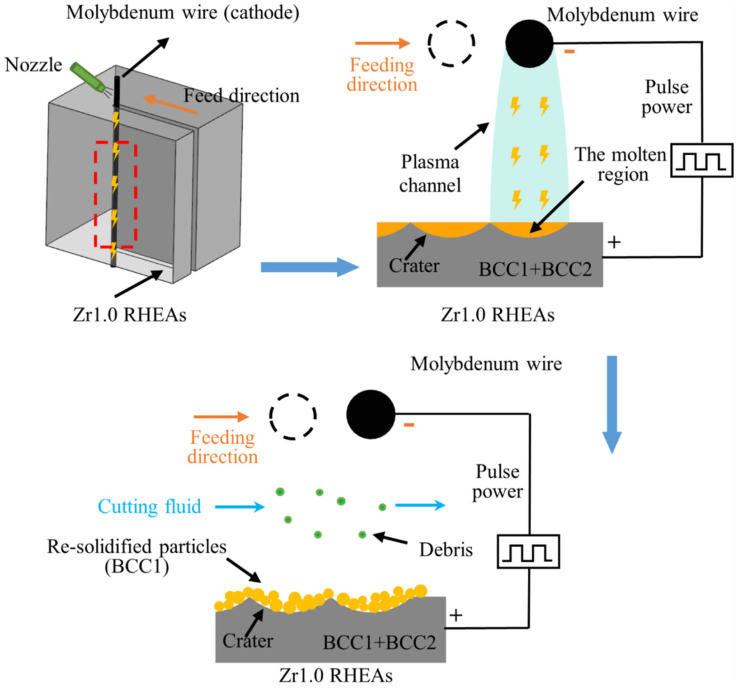
Schematic diagram showing the WEDM of the Zr1.0 RHEA.

Based on the above, the schematic diagram the WEDM process of the typical Zr1.0 RHEA is shown in Figure 11. With the feeding of the molybdenum wire, the plasma channel was formed, resulting in the melting of the workpiece materials. Part of the melted materials formed debris, which was removed by cutting fluid, while the other part of the melts re-solidified as spherical particles, forming the upper recast layer. During the WEDM, the BCC2 phase enriched with the Zr element was re-melted into liquid, and a large amount of the Zr element was removed by sparking (Table 6). This results in the significant decrease of the Zr content, where the BCC2 phase was therefore eliminated, leaving mainly the BCC1 phase for the upper recast layer. For most of the WEDM conditions in the present work, a large number of re-solidified particles were formed, and the upper recast layer resulted in a worse surface quality after the WEDM. The uncovering of the detailed mechanisms for the formation of the upper recast layer for these RHEAs is worthy of further investigation, to further improve the WEDMed surface quality.

### 3.4. WEDM Results of the RHEAs, Based on the Single-Factor Experiments

Although the variations of the CE and the surface roughness for the WEDM of the RHEAs have been revealed in orthogonal experiments, the processing parameters varied from group to group, and only the limited ranges for the processing parameters were conducted. Based on the findings obtained by the orthogonal tests, the effects of the ON, OFF, IP and SV on the WEDM performance of the Zr0.5 and Zr1.0 RHEAs were further examined by the single-factor experiments with wider parameter ranges. The effects of the processing parameters on the CE and surface roughness (Ra) of the RHEAs are given below.

#### 3.4.1. Cutting Efficiency (CE)

**Figure 12:**
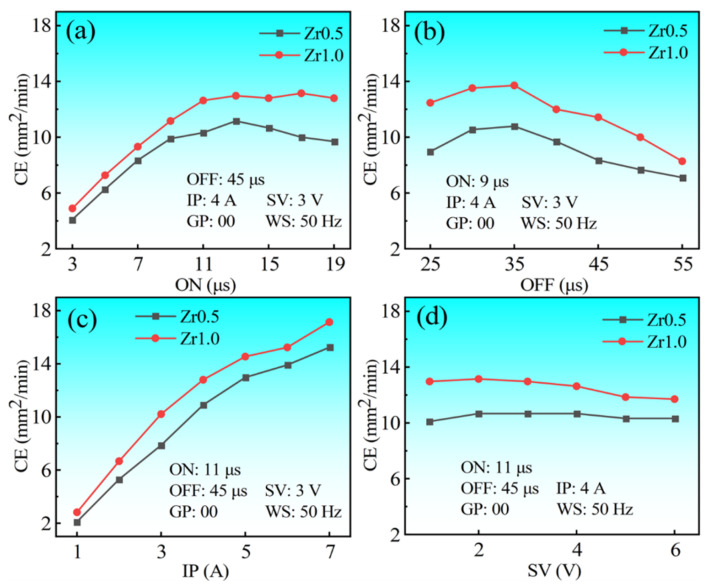
Effects of the processing parameters (ON, OFF, IP, and SV) on the CE of the Zr0.5 and Zr1.0 RHEAs: (**a**) ON; (**b**) OFF; (**c**) IP; and (**d**) SV.

The effects of the four parameters on the CE of the two RHEAs are shown in Figure 12. The two RHEAs exhibited similar trends on the increase of the four processing parameters, however, the CE of the Zr1.0 RHEA was higher than that of the Zr0.5 RHEA for all of the conditions. This is due to the fact that the Zr1.0 RHEA has a lower melting point (2952 K) than that of the Zr0.5 RHEA (3043 K). Under similar sparking conditions, more workpiece materials will be melted and removed. Looking into the variation trends in detail, the CE increased with the increase of the ON till the value of 11 μs (Figure 12a). With the increase of the ON, the duty-cycle of the discharge pulse also increased. The duty-cycle represents the percentage utilization of the pulse length in time. The increase of the duty-cycle will cause the increase of the discharge energy within a certain time period, further improving the CE [43,44]. However, there was no significant increase in the CE when the ON exceeded 13 μs. This may be due to the fact that with the further increase of the discharge energy, more debris were formed, which may affect the stability of the discharge process, reducing the CE. As shown in Figure 12b, the CE increased firstly and then decreased with the decrease of the OFF. When the pulse-off time was smaller than 35 μs, the decrease of the OFF caused the decrease of time spacing for the deionizing of the dielectric fluid, where the processing state became unstable, leading to the decrease of the CE. When the OFF was larger than 35 μs, the increase of the OFF led to the decrease of the discharge energy per unit time, thus resulting in the reduced CE. As shown in Figure 12c, the CE increased with the increase of the IP in the whole range, which is in agreement with the results of the orthogonal experiments. This can be explained by the fact that the increased IP can increase the discharge energy, benefiting the melting and removal of the workpiece materials. As shown in Figure 12d, the CE of the Zr0.5 and Zr1.0 RHEAs have no obvious variation with the increase of the SV. To conclude, the CE of the two RHEAs are significantly affected by the ON, OFF, and IP, rather than the SV. Thus, in order to tune the CE during the WEDM of the RHEAs, more attention should be paid to these three processing parameters.

#### 3.4.2. Surface Roughness (Ra)

**Figure 13:**
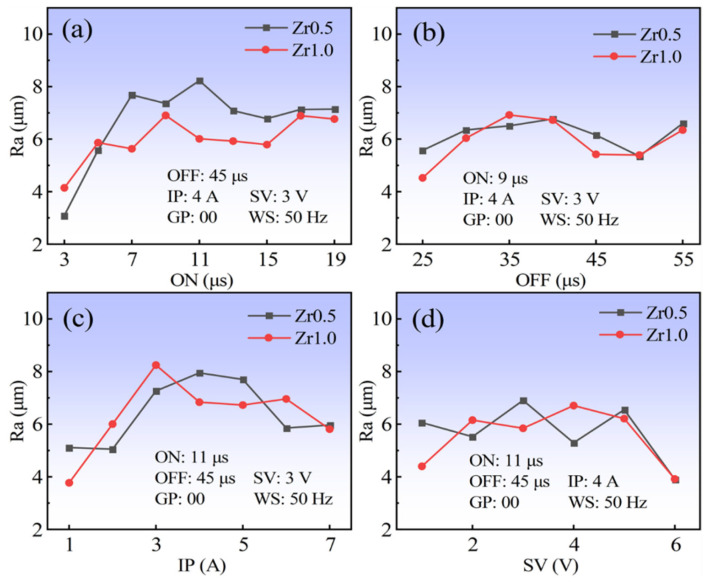
Effects of the processing parameters (ON, OFF, IP, and SV) on the surface roughness (Ra) of the Zr0.5 and Zr1.0 RHEAs: (**a**) ON; (**b**) OFF; (**c**) IP; and (**d**) SV.

**Figure 14:**
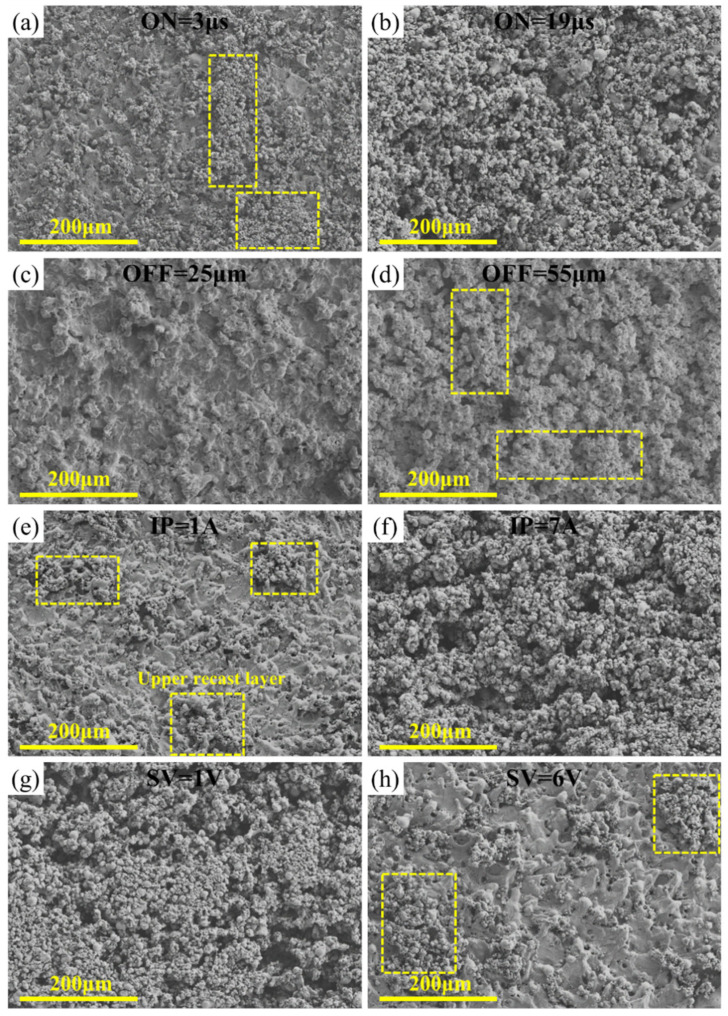
SEM images showing the WEDMed surface morphology of the Zr0.5 RHEA under different parameters, based on the single-factor experiments. (**a**,**b**) show the WEDMed surface morphology at ON = 3 μs and 19 μs, respectively; (**c**,**d**) show the WEDMed surface morphology at OFF = 25 μs and 55 μs, respectively; (**e**,**f**) show the WEDMed surface morphology at IP = 1A and 7A, respectively; and (**g**,**h**) show the WEDMed surface morphology at SV = 1V and 6V, respectively.

The effects of the ON, OFF, IP, and SV on the surface roughness (Ra) of the two RHEAs are shown in Figure 13. The two RHEAs also have similar trends with the increase of the ON, OFF, IP, and SV. However, differing from the CE results in Figure 12, there is no particularly significant difference for the surface roughness between the two RHEAs. As shown in Figure 13a, the Ra data increased firstly and then tended to become steady with the increase of the ON. Similarly, the Ra also increased initially with the increase of the IP, and then decreased with the further increase of the IP. The increase of the surface roughness with the increase of the ON and IP can be easily understood as the increase of discharge energy, where more workpiece materials were melted and re-solidified on the WEDMed surfaces, forming the recast layer with an enhanced surface roughness. While with the further increase of the discharge energy, the larger explosive forces may also be obtained. This will benefit the removal of the melted alloys, which may even reduce the surface roughness, for example, at the IP of 7 A (Figure 13c). As shown in Figure 14b, no significant variation trend with the increase of the OFF was observed. This may be due to the fact that the OFF mainly influenced the discharge intervals between pulses, which did not affect the discharge energy of a single pulse. The SV also has no significant effect on the change of the surface roughness, similar to the CE, except for the value of 6 V (Figure 13d). With the decrease of the ON and IP, the obvious decreasing trends in the Ra data were observed, and the lowest Ra of 3.1 μm for the Zr0.5 RHEA was achieved (Figure 13a). However, combined with the surface roughness data in the orthogonal tests (Figure 7 and Figure 8), it indeed showed that the relatively-high Ra data, ranging from 4 μm to 8 μm, were obtained for most of the groups (Figure 13). In this work, the optimum surface roughness value (3.1 μm) is lower than that of the machined surfaces produced by the first cut from the literature [30,31].

The morphology of the WEDMed surfaces of the typical Zr0.5 RHEA under different parameters is shown in Figure 14. It can be seen that the surface morphology has significant differences under different processing parameters. For all of the specimens with alarger surface roughness (Figure 14b,d,e), the WEDMed surface was covered by clusters of re-solidified particles, i.e., the upper recast layer. While for the specimens with a relatively lower surface roughness (Figure 14a,c,f), fewer clusters of the re-solidified particles were found, and the bottom of the recast layer can be clearly observed. Additionally, the surface roughness was also affected by the size of the re-solidified particles. Based on the above, in order to achieve a better surface roughness, more attention should be paid to reducing the amount of the upper recast layer, consisting of re-solidified particles, and the sizes of the particles, where the optimization of the ON, IP, and SV could be an effective route.

As a new class of difficult-to-cut structural materials with high strength and hardness, the processing of RHEAs is vital before their engineering applications in industry. By comparing the WEDM performance of the Zr0.5 and Zr1.0 RHEAs under varying processing parameters, the findings have shown that the CE can be easily tuned by optimizing the processing parameters, especially the ON, OFF, and IP. While it is challenging to tune the surface roughness by changing the processing parameters, although the decrease of the IP and OFF can contribute to the decrease of the Ra data. The findings have also demonstrated that the formation of the upper recast layers, consisting of clusters of particles on the WEDMed surfaces, are the main reasons for the relatively larger surface roughness of the RHEAs. Thus, more attention should be paid to reducing the upper recast layer, to achieve a better surface quality. The proportion of the upper recast layer consisting of spherical particles can be estimated by the SDE, and the increasing of the SDE is beneficial for reducing the number of spherical particles. For example, according to Equation (6), the improvement of the SV can effectively enhance the SDE value. The single-factor experiments have confirmed that the increase of the SV to 6 V can bring in a much smaller surface roughness. Moreover, the use of advanced WEDM techniques, such as the ultrasonic vibration assisted WEDM, can improve the removal of debris, which may also be helpful for achieving a better surface quality. Additionally, the RHEAs maintain the main BCC1 phase enriched with the W and Ta elements after the WEDM, while the BCC2 phase enriched with the Zr and Nb elements was removed. Further understanding of the melting and removal mechanisms of the RHEAs during the WEDM, for example, the formation of the particles and the change of the original phase structures, may play a critical role in improving the WEDM performance.

## 4. Conclusions

The WEDM performance of WNbMoTaZr*_x_* (*x* = 0.5, 1) RHEAs was investigated, and compared with pure tungsten, cemented carbide (YG8), and industrial pure Zr (Zr702), under both orthogonal and single-factor experiments. The CE, surface roughness, surface morphology, as well as the variation of the parent elements on the WEDMed surfaces, were studied and discussed. The main findings are given as below:

(1) According to the orthogonal experimental results, the GP was the most influencing factor for the CE of all kinds of materials, followed by the ON, IP, and OFF. The ON and IP were the two main contributors for the surface roughness of the Zr0.5 and Zr1.0 RHEAs and tungsten. The higher surface roughness mainly is attributed to the formation of the upper recast layer consisting of re-solidified particles, which was related to the specific discharge energy (SDE).

(2) The differences in the CE among the different workpiece materials was mainly attributed to the different melting point, while the surface roughness shows less dependence on the melting points. Based on the statistical analyses, the CE data of the RHEAs have relatively smaller Weibull moduli than that for the Ra data. The findings suggest that the CE of RHEAs can be tuned by optimizing the processing parameters, however, it is challenging to tune the surface roughness of RHEAs by tailoring the processing parameters.

(3) After WEDM, the RHEAs maintained the main BCC1 phase enriched with the W and Ta elements, which is different from the significant carbonization effect in the BMGs. While the BCC2 phase enriched with the Zr and Nb elements in the Zr1.0 RHEA disappeared. This can be further confirmed by the loss of the parent Zr element after the WEDM. The schematic diagram showing the WEDM process of the RHEAs was proposed and discussed.

(4) The effects of the processing parameters on the CE and the surface roughness of the RHEAs were further investigated and discussed, based on the single-factor experiments. The CE of the RHEAs was significantly affected by the ON, OFF, and IP, rather than the SV. While the surface roughness was mainly dependent on the ON and IP. Although the relatively lower surface roughness of about 3.1 μm was also observed for the Zr0.5 RHEA, most groups of RHEAs demonstrated relatively high Ra data, ranging from 4 μm to 8 μm.

(5) Strategies to further improve the surface quality of the WEDMed RHEAs were illustrated. The high SV can effectively improve the specific discharge energy (SDE), which is beneficial for the melting and throw-out of materials. This can effectively reduce the upper recast layer consisting of re-solidified particles. The low ON and IP can also improve the surface roughness effectively. Moreover, the ultrasonic vibration assisted WEDM may be useful to achieve a better surface quality by enhancing the removal of debris.

## Figures and Tables

**Table 1:** Composition of the Zr0.5 and Zr1.0 RHEAs, Zr702, tungsten and YG8 (at.%).

Materials	W	Nb	Mo	Ta	Zr	C	Hf	Co
Zr0.5	22.22	22.22	22.22	22.22	11.12	-	-	-
Zr1.0	20	20	20	20	20	-	-	-
Zr702	-	-	-	-	≥94.7	-	4.5	-
Tungsten	≥99.95	-	-	-	-	-	-	-
YG8 [33]	43.69	-	-	-	-	43.69	-	12.62

**Table 2:** Melting points and the thermal conductivity of the five workpiece materials [32,34,35].

Materials	Zr0.5	Zr1.0	Tungsten	YG8	Zr702
Melting points (K)	3043	2952	3695	3196	2128
Thermal conductivity/W·(m·K)^−1^	26	25	175	70	22

**Table 3:** Processing parameters for the orthogonal WEDM experiments.

Group	Pulse-On Time/μs	Pulse-Off Time/μs	Peak Current/A	Servo Voltage/V	Pulse Type	Barrel Running Frequency (Hz)
1	6	35	3	2	00	20
2	6	45	4	3	01	40
3	6	55	5	4	02	50
4	9	35	3	3	01	50
5	9	45	4	4	02	20
6	9	55	5	2	00	40
7	12	35	4	2	02	40
8	12	45	5	3	00	50
9	12	55	3	4	01	20
10	6	35	5	4	01	40
11	6	45	3	2	02	50
12	6	55	4	3	00	20
13	9	35	4	4	00	50
14	9	45	5	2	01	20
15	9	55	3	3	02	40
16	12	35	5	3	02	20
17	12	45	3	4	00	40
18	12	55	4	2	01	50

**Table 4:** The WEDM CE (mm^2^/min) results of the five workpiece materials (Zr0.5 and Zr1.0 RHEAs, Zr702, pure tungsten and YG8).

Group	Zr0.5	Zr1.0	Zr702	Tungsten	YG8
1	7.62	9.50	12.97	2.98	9.60
2	3.89	4.47	5.75	1.56	3.30
3	2.62	3.18	4.00	1.33	1.50
4	4.62	5.61	8.35	2.01	5.19
5	3.74	4.25	4.44	1.66	3.24
6	11.71	14.55	19.20	5.30	12.31
7	6.04	7.44	12.31	2.21	6.58
8	14.12	18.11	25.26	6.58	16.55
9	2.44	3.65	4.64	1.42	1.29
10	5.58	5.85	7.50	2.28	4.75
11	2.22	3.20	4.09	0.92	2.41
12	6.67	7.38	10.00	3.12	5.75
13	12.80	14.33	21.82	5.11	13.15
14	5.71	7.93	10.43	2.76	6.23
15	2.56	3.10	4.75	1.18	2.71
16	7.74	8.89	13.71	3.09	8.07
17	8.73	10.79	17.78	3.58	11.16
18	4.42	5.11	8.42	2.25	3.82

**Table 5:** The WEDM Ra (μm) results of the five materials: Zr0.5, Zr1.0, Zr702, pure tungsten, and YG8.

Group	Zr0.5	Zr1.0	Zr702	Tungsten	YG8
1	4.55	4.84	4.07	2.28	1.47
2	6.00	4.86	5.14	2.86	1.32
3	5.35	5.16	4.72	3.01	1.45
4	6.21	5.59	5.76	2.30	1.58
5	6.45	5.38	4.95	3.03	1.44
6	7.13	5.11	5.23	3.45	2.79
7	6.21	6.17	6.94	3.05	3.65
8	7.50	7.27	6.30	3.59	2.69
9	4.41	4.18	6.20	3.22	1.53
10	6.33	5.46	4.34	3.11	1.38
11	5.11	4.77	4.78	2.52	1.60
12	4.70	4.64	5.26	2.43	1.36
13	4.54	6.14	4.83	3.30	1.75
14	5.69	5.87	6.59	3.26	2.41
15	6.08	6.38	5.21	2.30	1.79
16	7.03	7.92	6.77	3.80	3.33
17	5.55	6.90	5.68	2.67	1.88
18	6.12	5.48	6.75	3.35	2.05

**Table 6:** The element content of the upper recast layer on the WEDMed surfaces of the typical Zr0.5 and Zr1.0 RHEAs (at.%).

Alloys	W	Nb	Mo	Ta	Zr
Zr0.5 (theoretical value)	22.22	22.22	22.22	22.22	11.12
Zr0.5 (group 1)	25.6 ± 2.1	20.0 ± 1.4	25.8 ± 4.7	25.1 ± 3.0	3.5 ± 2.6
Zr0.5 (group 13)	26.1 ± 1.7	19.8 ± 1.2	28.1 ± 2.0	21.5 ± 0.4	4.6 ± 3.8
Zr1.0 (theoretical value)	20	20	20	20	20
Zr1.0 (group 1)	26.6 ± 1.8	16.8 ± 1.4	26.8 ± 3.2	25.0 ± 3.8	5.0 ± 1.4
Zr1.0 (group 13)	26.5 ± 1.2	21.3 ± 2.8	27.1 ± 1.2	21.7 ± 1.3	3.4 ± 1.5

## Data Availability

The raw/processed data will be made available on request.

## Appendix A

**Table A1:** ANOVA for the CE of the five materials.

Material	Parameters	DF	Adjusted	Contribution (%)	Adjusted MS	F-Value	Rank
Zr0.5	ON	2	21.360	9.65	10.680	12.96	3
	OFF	2	16.398	7.41	8.199	9.95	4
	IP	2	31.017	14.02	15.509	18.82	2
	SV	2	1.135	0.52	0.567	0.69	6
	GP	2	143.134	64.69	71.567	86.87	1
	WS	2	4.091	1.85	2.046	2.48	5
	Error	5	4.119	1.86	0.824	-	-
	Total	17	221.254	100.00	-	-	-
Zr1.0	ON	2	38.652	11.76	19.326	13.26	3
	OFF	2	20.133	6.12	10.066	6.91	4
	IP	2	44.764	13.62	22.382	15.36	2
	SV	2	3.490	1.06	1.745	1.20	6
	GP	2	209.106	63.61	104.553	71.76	1
	WS	2	5.283	1.61	2.641	1.81	5
	Error	5	7.285	2.22	1.457	-	-
	Total	17	328.713	100.00	-	-	-
Zr702	ON	2	122.882	17.06	61.441	32.59	2
	OFF	2	56.531	7.85	28.266	14.99	4
	IP	2	64.639	8.97	32.320	17.14	3
	SV	2	6.174	0.86	3.087	1.64	6
	GP	2	438.961	60.93	219.481	116.42	1
	WS	2	21.772	3.02	10.886	5.77	5
	Error	5	9.426	1.31	1.885	-	-
	Total	17	720.385	100.00	-	-	-
Tungsten	ON	2	4.636	11.24	2.318	13.75	3
	OFF	2	0.882	2.14	0.441	2.62	4
	IP	2	7.186	17.43	3.593	21.31	2
	SV	2	0.388	0.94	0.194	1.15	6
	GP	2	26.438	64.11	13.219	78.41	1
	WS	2	0.865	2.10	0.432	2.56	5
	Error	5	0.843	2.04	0.169	-	-
	Total	17	41.238	100.00	-	-	-
YG8	ON	2	37.167	11.28	18.584	31.56	2
	OFF	2	36.581	11.11	18.290	31.07	3
	IP	2	27.043	8.21	13.522	22.97	4
	SV	2	4.247	1.29	2.123	3.61	6
	GP	2	214.828	65.22	107.414	182.44	1
	WS	2	6.583	2.00	3.291	5.59	5
	Error	5	2.944	0.89	0.589	-	-
	Total	17	329.393	100.00	-	-	-

**Table A2:** ANOVA for the surface roughness (Ra) of the five materials.

Material	Parameters	DF	Adjusted	Contribution (%)	Adjusted MS	F-Value	Rank
Zr0.5	ON	2	2.207	15.25	1.103	1.74	2
	OFF	2	0.529	3.65	0.264	0.42	5
	IP	2	4.450	30.76	2.225	3.51	1
	SV	2	1.988	13.75	0.994	1.57	3
	GP	2	0.441	3.05	0.220	0.35	6
	WS	2	1.687	11.66	0.843	1.33	4
	Error	5	3.166	21.88	0.633	-	-
	Total	17	14.468	100.00	-	-	-
Zr1.0	ON	2	5.619	36.63	2.809	5.88	1
	OFF	2	1.489	9.71	1.245	2.60	2
	IP	2	1.902	12.40	0.951	1.99	3
	SV	2	1.801	11.74	0.900	1.88	4
	GP	2	1.755	11.44	0.877	1.84	5
	WS	2	0.383	2.49	0.191	0.40	6
	Error	5	2.390	15.59	0.478	-	-
	Total	17	15.339	100.00	-	-	-
Zr702	ON	2	8.956	67.63	4.478	21.05	1
	OFF	2	0.054	0.41	0.027	0.13	6
	IP	2	0.534	4.04	0.267	1.26	4
	SV	2	1.508	11.39	0.754	3.54	2
	GP	2	0.984	7.43	0.492	2.31	3
	WS	2	0.143	1.08	0.072	0.34	5
	Error	5	1.064	8.02	0.213	-	-
	Total	17	13.243	100.00	-	-	-
Tungsten	ON	2	1.006	27.14	0.503	5.00	2
	OFF	2	0.003	0.07	0.001	0.01	6
	IP	2	2.042	55.05	1.021	10.14	1
	SV	2	0.098	2.63	0.049	0.48	3
	GP	2	0.017	0.45	0.008	0.08	5
	WS	2	0.040	1.09	0.020	0.20	4
	Error	5	0.503	13.57	0.101	-	-
	Total	17	3.709	100.00	-	-	-
YG8	ON	2	3.568	41.62	1.782	27.38	1
	OFF	2	0.460	5.37	0.230	3.53	5
	IP	2	1.487	17.34	0.743	11.41	3
	SV	2	1.731	20.19	0.865	13.28	2
	GP	2	0.748	8.72	0.374	5.74	4
	WS	2	0.254	2.96	0.127	1.95	6
	Error	5	0.326	3.80	0.065	-	-
	Total	17	8.574	100.00	-	-	-
